# Results of a national school-based deworming programme on soil-transmitted helminths infections and schistosomiasis in Kenya: 2012–2017

**DOI:** 10.1186/s13071-019-3322-1

**Published:** 2019-02-07

**Authors:** Charles Mwandawiro, Collins Okoyo, Jimmy Kihara, Elses Simiyu, Stella Kepha, Suzy J. Campbell, Matthew C. Freeman, Simon J. Brooker, Sammy M. Njenga

**Affiliations:** 1 Eastern and Southern Africa Centre of International Parasite Control, Kenya Medical Research Institute (KEMRI), Nairobi, Kenya; 2 Faculty of Infectious and Tropical Diseases, London School of Hygiene and Tropical Medical Medicine, Keppel Street, London, WC1E 7HT UK; 3 Pwani University Biosciences Research Centre (PUBRec), Pwani University, Kilifi, Kenya; 4 Evidence Action, Washington DC, USA; 5 Department of Environmental Health, Rollins School of Public Health, Emory University, Atlanta, GA USA

**Keywords:** School-based deworming, Soil-transmitted helminths, Schistosomiasis

## Abstract

**Background:**

Soil-transmitted helminth (STH) and schistosome infections are among the most prevalent neglected tropical diseases (NTDs) in the world. School-aged children are particularly vulnerable to these chronic infections that can impair growth, nutritional status and cognitive ability. Mass drug administration (MDA) delivered either once or twice annually is a safe and effective approach recommended by the World Health Organization (WHO) to reduce worm burden. In 2012, Kenya began a national school-based deworming programme (NSBDP) aimed at reducing infection and associated morbidity. The change in prevalence and intensity of these infections was monitored over five years (2012–2017). Here, we present the changes in STH and schistosome infections between baseline and endline assessments, as well as explore the yearly patterns of infection reductions.

**Methods:**

We used series of pre- and post-MDA intervention, repeat cross-sectional surveys in a representative, stratified, two-stage sample of schools in 16 counties of Kenya. The programme consisted of two tiers of monitoring; a national baseline, midterm and endline surveys consisting of 200 schools, and pre- and post-MDA surveys conducted yearly consisting of 60 schools. Stool and urine samples were collected from randomly selected school children and examined for STH and schistosome infections using Kato-Katz and urine filtration techniques respectively.

**Results:**

Overall, 32.3%, 16.4% and 13.5% of the children were infected with any STH species during baseline, midterm and endline assessment, respectively, with a relative reduction of 58.2% over the five-year period. The overall prevalence of *S. mansoni* was 2.1%, 1.5% and 1.7% and of *S. haematobium* was 14.8%, 6.8% and 2.4%, respectively, for baseline, midterm and endline surveys. We observed inter-region and inter-county heterogeneity variation in the infection levels.

**Conclusions:**

The analysis provided robust assessment of the programme and outlined the current prevalence, mean intensity and re-infection pattern of these infections. Our findings will allow the Government of Kenya to make informed decisions on the strategy to control and eliminate these NTDs. Our results suggest that complimentary interventions may have to be introduced to sustain the chemotherapeutic gains of MDA and accelerate attainment of elimination of these NTDs as a public health problem in Kenya.

**Electronic supplementary material:**

The online version of this article (10.1186/s13071-019-3322-1) contains supplementary material, which is available to authorized users.

## Background

Soil-transmitted helminths (STH: *Ascaris lumbricoides*, *Trichuris trichiura*, and the hookworms, *Necator americanus* and *Ancylostoma duodenale*) and schistosomes (*Schistosoma mansoni* and *Schistosoma heamatobium*) are among the diseases classified by the World Health Organization (WHO) as neglected tropical diseases (NTDs) [1]. STH infections are endemic in 166 countries worldwide [2] while schistosome infections are endemic in 76 countries [3], and these infections combined affect more than three billion people globally and occur mainly in sub-Saharan Africa, the Americas, China and East Asia with the burden of prevention and control of these infections costing developing economies billions of dollars every year [4]. For this reason, WHO has prioritized both STH and schistosome infections for elimination by the year 2020 [5], through delivery of mass drug administration (MDA) to at-risk populations, including pre-school and school-aged children [6]. Majority of these countries which are endemic for both STH and schistosome infections are now implementing MDA programmes, either through school-based deworming (SBD) or lymphatic filariasis control programmes [7, 8].

STH infections are caused by ingestion of eggs from contaminated soil (*A. lumbricoides* and *T. trichiura*) or by active penetration of the skin by larvae in the soil (hookworms). Infected people depict a wide range of symptoms that may include nausea, tiredness, abdominal pain, and loss of appetite that is likely to aggravate malnutrition and amplify rates of anaemia. In children, these infections impede physical growth and cognitive development, contributing significantly to school absenteeism [9]. Human schistosomiasis, commonly known as bilharzia, is a water-based parasitic infection caused by blood flukes (trematodes) [10]. Adult schistosome worms invade human blood vessels and the immune system while excreting hundreds to thousands of eggs daily. Trapped eggs induce a distinct immune-mediated granulomatous response that causes local and systemic pathological effects ranging from anaemia, growth stunting, impaired cognition, decreased physical fitness and organ-specific effects like severe hepatosplenomegaly, periportal fibriosis with portal hypertension and urogenital inflammation and scarring [10].

At present, preventive public health measures in endemic countries consist of treatment once or twice annually, depending on the risk profile of the area, with albendazole or mebendazole for STH infections, and praziquantel for schistosome infections. Preventive chemotherapy to all at-risk children is often delivered through SBD programmes, which offers benefits to the treated children, overall population, and cost savings for programme implementers. Nonetheless, SBD programmes do not prevent re-infections which can occur rapidly after treatment; as such, in many contexts there is need for environmental improvements, specifically access to improved water and sanitation, as well as hygiene behaviours (WASH) to maximize on the benefit of preventive chemotherapy [11, 12].

The Kenyan Ministries of Health (MoH) and Education (MoE) began a National School Based Deworming Programme (NSBDP) in the year 2012 in 66 sub-counties endemic for both STH and schistosome infections in four regions (Western, Nyanza, Rift Valley and Coast). In 2011, a National School Health Policy and National Multi-Year Strategic Plan for the Control of NTDs were developed and called for treatment to be administered to all school-aged children, including those out of school, based on the prevalence and intensity of STH and schistosome infections in these regions in order to reduce infections to a level where they are no longer a public health problem (i.e. prevalence of moderate to heavy infections to below 1%). The strategic plan informs a comprehensive strategy for the integration of NTD control efforts [12, 13].

The impact of the Kenyan NSBDP on STH and schistosome infections was monitored from 2012 to 2017 using an extensive monitoring and evaluation (M&E) programme led by the Kenya Medical Research Institute (KEMRI) that included pre- and post-MDA intervention and repeated cross-sectional surveys as indicated in Additional file 1: Figure S1. Baseline and midterm results for this M&E programme were provided by Mwandawiro et al. [13] and Okoyo et al. [12], respectively.

Our present analysis, involves the five-year findings of this M&E programme in the 200 schools surveyed for baseline, midterm and endline assessments and in 60 schools surveyed yearly from 2012 to 2017. The specific aims of this study were to determine the overall reductions in prevalence and intensity of infections achieved from baseline to endline, the yearly patterns of treatment impact, and impact on moderate-to-heavy intensity of infections.

## Methods

### Study design

The M&E of the NSBD programme included a series of pre- and post-intervention, repeat cross-sectional surveys in a representative, stratified, two-stage sample of schools across several regions in Kenya. Sub-county stratification was based on both geography and anticipated infection prevalence. There were two tiers of monitoring: (i) a national baseline, midterm (after two MDA rounds) and endline survey (after four MDA rounds); these surveys included 200 schools in 20 sub-counties from 16 counties that aimed to establish an accurate national measurement of infection levels; and (ii) surveys conducted in 60 out of the 200 schools before and 3–5 weeks after treatment (pre-post surveys) to evaluate the immediate reductions in infections that can be directly attributed to the programme implementation (Additional file 1: Figure S1).

A sample size of 200 schools with approximately 108 children per school for the baseline, midterm and endline assessment was determined to be adequate to detect a 5% change in prevalence and intensity of infection from baseline to endline at a national level, assuming a power of 80% and test size of 5%, and considering the anticipated variance in prevalence [12, 13]. The 200 schools were selected randomly prior to baseline survey from 66 sub-counties based on the geographical distribution of the population and the infections endemicity from the available data and predictive maps [14, 15]. The sub-counties were further grouped into infection level strata and 20 sub-counties from 16 counties randomly selected in the first sampling stage, with the number of sub-counties per region proportional to the population. At the second sampling stage, primary schools were randomly selected from within the chosen 20 sub-counties. In each school, 18 children (9 girls and 9 boys) were sampled randomly from each of the six classes; one early childhood development (ECD) class and classes 2–6 using random number tables, for a total of approximately 108 children per school (Table 1). A detailed description of this M&E programme design is provided elsewhere [12, 13].

**Table 1:** Number of schools and children examined by county among Kenyan school-aged children, 2012–2017

County	Year 1		Year 2		Year 3		Year 4		Year 5	
	Baseline	Y1 Post-MDA	Y2 Pre-MDA	Y2 Post-MDA	Midterm	Y3 Post-MDA	Y4 Pre-MDA	Y4 Post-MDA	Endline	Y5 Post-MDA
Bomet	12 (1296)	3 (324)	3 (313)	3 (319)	12 (1298)	3 (313)	3 (316)	3 (323)	12 (1296)	3 (319)
Bungoma^a^	9 (968)	2 (216)	2 (215)	2 (216)	9 (935)	2 (203)	2 (207)	2 (205)	9 (944)	2 (214)
Busia	18 (1942)	6 (648)	6 (641)	6 (643)	18 (1927)	6 (647)	6 (643)	6 (626)	18 (1916)	6 (637)
Homa Bay^b^	24 (2590)	6 (642)	6 (646)	6 (634)	24 (2483)	6 (631)	6 (635)	–^c^	23 (2458)	6 (628)
Kakamega	20 (2152)	6 (648)	6 (641)	6 (644)	20 (2086)	6 (608)	6 (623)	6 (618)	20 (2108)	6 (637)
Kericho	12 (1292)	3 (324)	3 (312)	3 (279)	12 (1297)	3 (295)	3 (315)	3 (321)	12 (1278)	3 (320)
Kilifi	10 (1080)	3 (316)	3 (324)	3 (324)	10 (1069)	3 (315)	3 (322)	3 (312)	10 (1040)	3 (307)
Kisii	10 (1296)	3 (324)	3 (320)	3 (318)	12 (1265)	3 (317)	3 (320)	3 (323)	12 (1264)	3 (318)
Kisumu	10 (1078)	3 (324)	3 (295)	3 (313)	10 (1032)	3 (323)	3 (323)	–^c^	10 (1069)	3 (316)
Kwale	18 (1940)	6 (642)	6 (648)	6 (648)	18 (1884)	6 (563)	6 (643)	6 (621)	18 (1857)	6 (615)
Migori	8 (864)	3 (226)	3 (323)	3 (314)	8 (863)	3 (314)	3 (317)	–^c^	8 (834)	3 (312)
Mombasa	8 (852)	3 (313)	3 (324)	3 (324)	8 (844)	3 (311)	3 (278)	3 (289)	8 (850)	3 (315)
Narok	10 (1070)	3 (324)	3 (322)	3 (274)	10 (1062)	3 (311)	3 (323)	3 (324)	10 (1054)	3 (322)
Nyamira	10 (1080)	3 (324)	3 (321)	3 (320)	10 (1073)	3 (313)	3 (301)	3 (322)	10 (1061)	3 (318)
Taita Taveta	10 (1072)	3 (311)	3 (318)	3 (324)	10 (1068)	3 (322)	3 (319)	3 (318)	10 (1058)	3 (277)
Vihiga	8 (860)	3 (324)	3 (319)	3 (320)	8(825)	3 (311)	3 (304)	3 (312)	8 (854)	3 (319)
Total	199 (21,432)	59 (6230)	59 (6282)	59 (6214)	199 (21,011)	59 (6097)	59 (6189)	47 (4914)	198 (20,941)	59 (6174)

^a^One school was replaced in Bungoma County during year 1 surveys and was therefore excluded together with the replacement school

^b^One school in Homa Bay County was not surveyed during year 5 endline survey since the school had been closed down at the time of that follow-up survey

^c^Surveys were not conducted during year 4 post-MDA in the three counties of Homa Bay, Kisumu and Migori

### Data collection

**Fig. 1:**
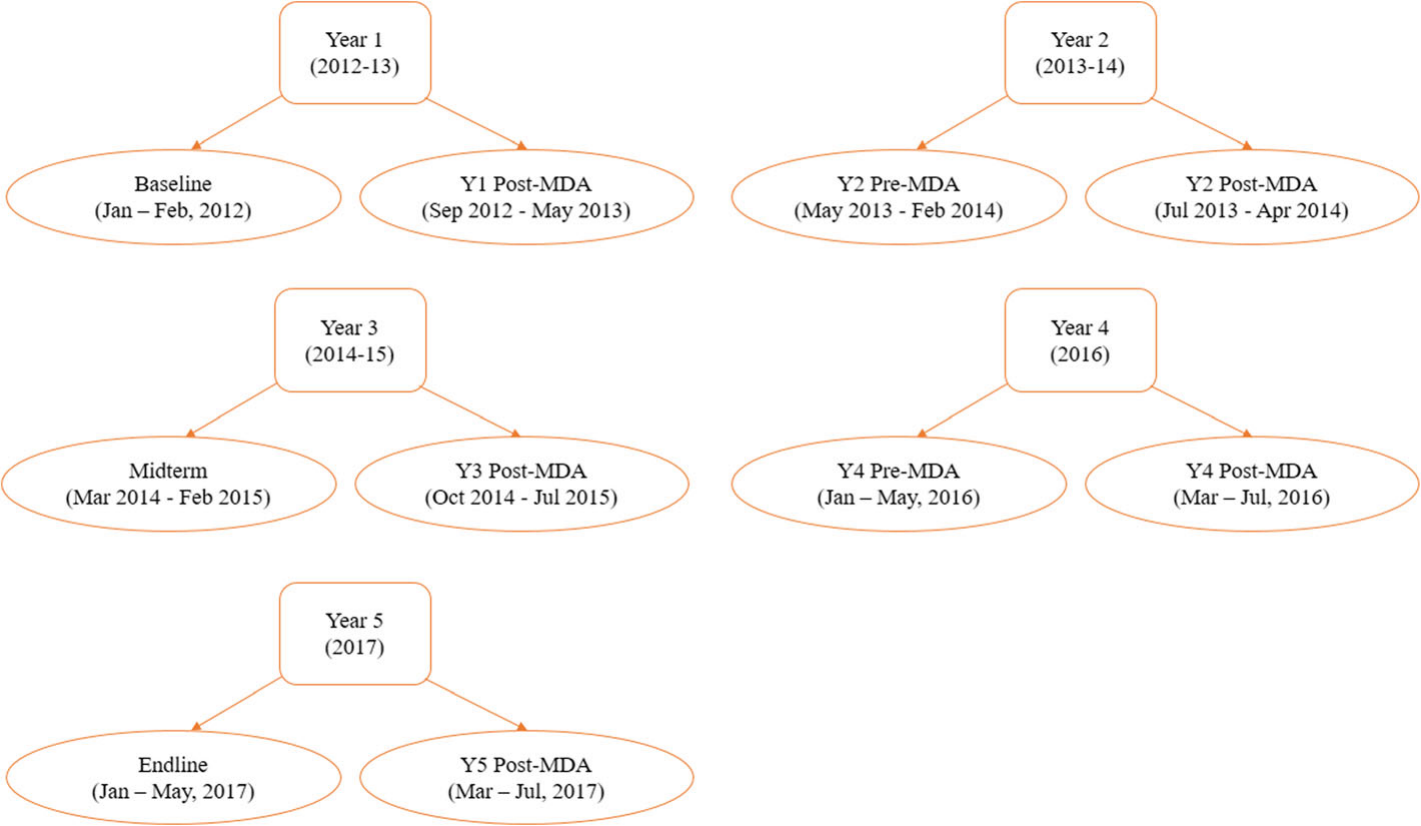
Data collection time points for the M&E programme, 2012–2017

The data collection time points for the M&E programme is shown in Fig. 1. In each of the years, pre-MDA surveys were carried out approximately one year after the previous year’s MDA delivery while post-MDA surveys were carried out approximately 12–47 days after that year’s MDA delivery to all school children by the NSBD programme. Laboratory data reporting form was programmed onto android-based smartphones and used to capture data electronically into the Open Data Kit (ODK) system [16] that incorporated in-built data quality checks to prevent data entry errors.

### Survey procedures

During each survey round, selected schools were visited one week prior to the survey date to explain the purpose of the survey to the school head teachers and school committees. Permission to conduct the survey was sought at school-level. Parental consent from parents/guardians of children in ECD class and those from classes 2–6 was based on passive, opt-out consent rather than written opt-in consent due to the low risk and routine nature of the survey.

#### Stool sample processing using Kato-Katz technique

Selected children were asked to provide stool samples which were processed in the laboratory within 24 h and examined in duplicate for the presence of STH and *S. mansoni* eggs by two technicians using the Kato-Katz thick smear technique that included a sieve and calibrated template of 41.7 mg. Eggs were counted in the entire cellophane film, then expressed as eggs per gram (epg) by multiplying with a standard conversion factor of 24. Any resulting discrepancies in the readings were resolved by a third senior technician. The laboratory results were recorded as mean number of eggs per gram of stool for each child, which is a commonly accepted proxy of infection intensity, an arguably more important public health measure than prevalence [17].

#### Urine sample processing using filtration technique

Urine samples were obtained only from children in the four counties (Kwale, Kilifi, Mombasa and Taita Taveta) of the Coastal Region where *S. haematobium* is focally prevalent. The collected urine samples were processed using the urine filtration technique in the laboratory within 24 h using the polycarbonate membrane filters and examined in duplicate for the presence of *S. haematobium* eggs by two technicians. Any resulting discrepancies in the readings were resolved by a third senior technician. The laboratory results were recorded as mean number of eggs per 10 ml of urine.

For quality control purposes, 10% of both stool and urine samples were randomly re-examined by a senior technologist. Both stool and urine samples were collected between 09:00 and 12:00 h each day of the survey. As part of the NSBD programme, all participating children were treated with albendazole (400 mg) for STH infections and praziquantel (40 mg/kg) for schistosome infections according to WHO guidelines [18].

### Statistical analysis

The prevalence of each helminth species and STH combined was calculated at the school and county level and 95% confidence intervals (CIs) obtained using binomial regression models accounting for the clustering at school level. Mean infection intensity was expressed as eggs per gram of faeces (epg) and 95% CIs obtained using negative binomial regression models accounting for the clustering at school level. Infection intensities were further classified as light, moderate and heavy infections, according to WHO guidelines and the prevalence of each infection class together with their 95% CIs obtained using binomial regression adjusting for school clusters. The relative reductions in prevalence and mean intensity of each STH species and light to heavy infections from baseline to endline surveys were calculated using multivariable mixed effects models with random intercepts for schools and counties and *P*-values obtained using Wald test. Both children and schools treatment coverage was determined by dividing the number of children and/or schools treated with either albendazole or praziquantel drug and the number targeted in a particular year. All statistical analyses were carried out using STATA version 14.1 (STATA Corporation, College Station, TX, USA). Graphs were developed using the *ggplot* package implemented in R [19]. School locations were mapped using ArcGIS Desktop version 10.2.2 software (Environmental Systems Research Institute Inc., Redlands, CA, USA).

## Results

Overall 200 schools (21,528 children) during baseline, 200 schools (21,111 children) during midterm and 199 schools (21,045 children) during endline were included in the assessments across 16 counties in Western, Nyanza, Rift Valley and Coast regions. Additionally, 60 schools (6300 children) were included in pre- and post-MDA assessments of each year. One school in Bungoma County was replaced after the baseline survey and was therefore excluded together with the replacement school to allow for comparability between baseline and follow-up surveys. During year 4 post-MDA surveys, 12 schools from 3 counties were not surveyed due to logistical challenges. Additionally, one school in Homa Bay County was not surveyed during year 5 endline survey since the school had been closed down at the time of that follow-up survey. Hence, the final analysis was conducted on 199 schools for baseline and midterm surveys, and 198 schools for endline survey and 59 schools for pre- and post-MDA surveys each year except year 4 post-MDA where analysis was conducted in 47 schools. The number of schools and children included in the final analysis per county at each time point is shown in Table 1. The mean age of children was 9.9 years and ranged from 2 to 24 years (standard deviation 2.6 years) with 50.1% being males. Children absent on the day of the survey were not included in the study.

### STH infections

**Fig. 2:**
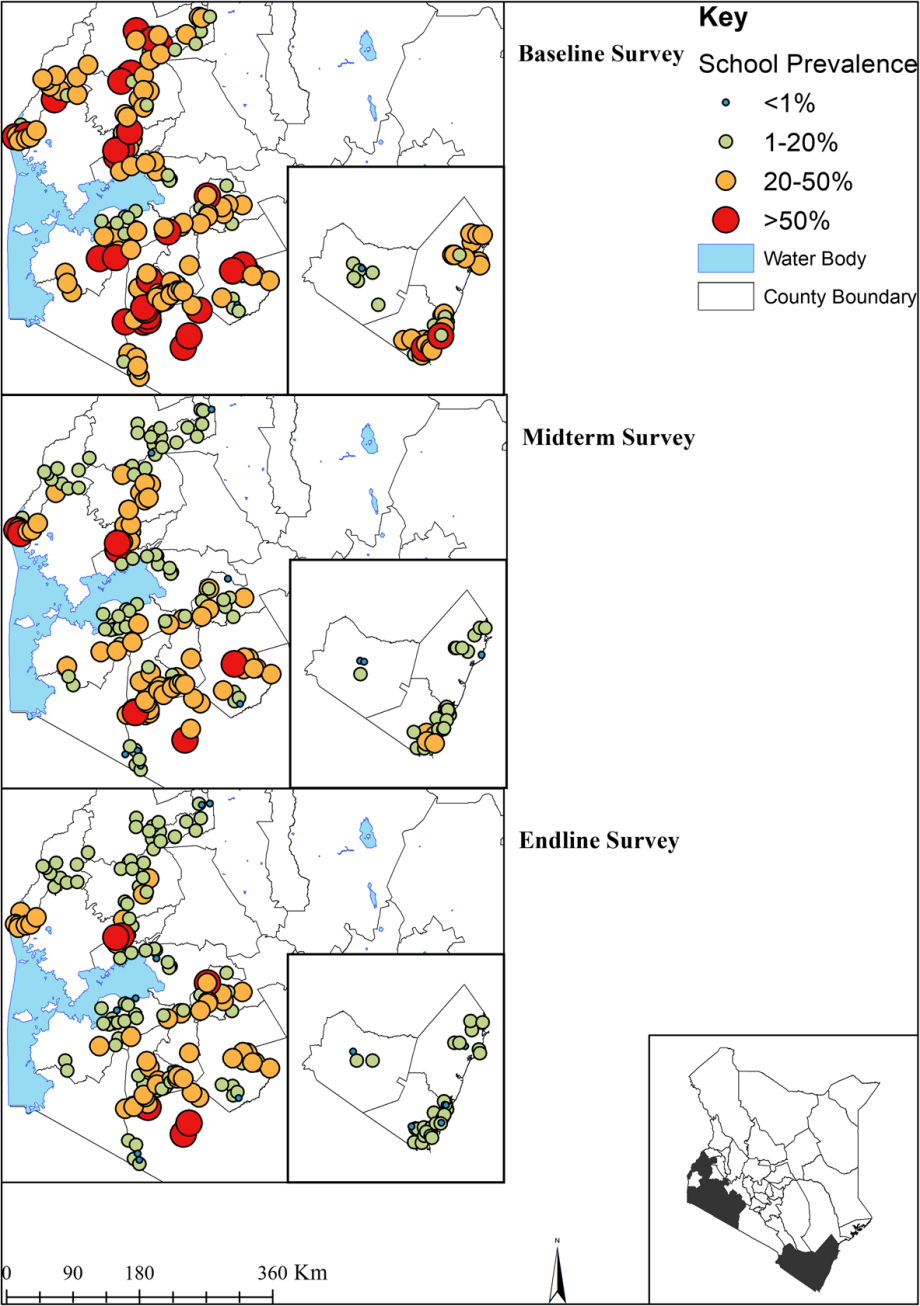
The geographical distribution of STH infections prevalence at baseline (2012), midterm (2015) and endline (2017) among Kenyan school-aged children

Figure 2 provides the geographical distribution of STH prevalence from baseline to endline. Overall, 32.3% (95% CI: 30.0–34.8%) of children were infected with at least one STH species during baseline, 16.4% (95% CI: 14.4–18.6%) during midterm and 13.5% (95% CI: 11.6–15.7%) during endline. This reduction amounted to a significant relative reduction (RR) of 58.2% (Wald test: *Z* = -14.20, *P* < 0.001) over the five-year period. *Ascaris lumbricoides* was the most prevalent STH species during all the surveys (baseline 18.1%, midterm 11.9% and endline 9.6%; RR 46.8%) followed by *T. trichiura* (baseline 6.7%, midterm 4.5% and endline 4.1%; RR 38.4%) and then hookworm (baseline 15.4%, midterm 2.3% and endline 1.3%; RR 91.6%). The overall mean intensity of *A. lumbricoides* was 1659 epg (95% CI: 1378–1998) during baseline, 960 epg (95% CI: 801–1151) during midterm and 917 epg (95% CI: 750–1121) during endline with a RR of 44.7% (Wald test: *Z* = -8.27, *P* < 0.001) (Table 2).

**Table 2:** Baseline, midterm and endline prevalence, mean intensity of infection and relative reductions % (Wald test: *Z*-statistic, *P*-value) among Kenyan school-aged children, 2012–2017

	Baseline	Midterm	Endline	Relative reduction (Baseline – Endline)
Survey prevalence, % (95% CI)				
STH Combined	32.3 (30.0–34.8)	16.4 (14.4–18.6)	13.5 (11.6–15.7)	58.2 (*Z* = -1.20, *P* < 0.001)
Hookworm	15.4 (13.6–17.6)	2.3 (1.8–3.0)	1.3 (1.0–1.6)	91.6 (*Z* = -21.34, *P* < 0.001)
*A. lumbricoides*	18.1 (15.8–20.7)	11.9 (10.2–13.9)	9.6 (8.0–11.5)	46.8 (*Z* = -10.82, *P* < 0.001)
*T. trichiura*	6.7 (5.4–8.2)	4.5 (3.4–6.0)	4.1 (3.1–5.5)	38.4 (*Z* = -5.38, *P* < 0.001)
*S. mansoni*	2.1 (1.3–3.5)	1.5 (0.7–3.1)	1.7 (1.0–2.8)	19.3 (*Z* = -1.86, *P* = 0.062)
*S. haematobium*	14.8 (11.3–19.5)	6.8 (4.3–10.7)	2.4 (1.3–4.3)	84.0 (*Z* = -5.74, *P* < 0.001)
Mean intensity, epg (95% CI)				
STH Combined	1756 (1472–2094)	985 (824–1177)	944 (774–1151)	46.3 (*Z* = -9.17, *P* < 0.001)
Hookworm	63 (50–81)	8 (5–14)	10 (5–19)	84.2 (*Z* = -5.58, *P* < 0.001)
*A. lumbricoides*	1659 (1378–1998)	960 (801–1151)	917 (750–1121)	44.7 (*Z* = -8.27, *P* < 0.001)
*T. trichiura*	33 (11–105)	17 (11–26)	16 (10–26)	50.9 (*Z* = -1.15, *P* < 0.001)
*S. mansoni*	12 (4–36)	5 (2–14)	5 (2–9)	61.6 (*Z* = -3.10, *P* = 0.002)
*S. haematobium*	16 (10–26)	7 (4–12)	2 (1–5)	87.2 (*Z* = -4.18, *P* < 0.001)

After five years of MDA implementation, Coast Region had 87.6% (Wald test: *Z* = -20.24, *P* < 0.001) lower STH infection prevalence between (24.2%) and endline (3.0%). Western Region had 60.6% (Wald test: *Z* = -21.46, *P* < 0.001) reduction and Nyanza had 59.4% (Wald test: *Z* = -20.55, *P* < 0.001) reduction. In Rift Valley any STH infections reduced by 27.5% (Wald test: *Z* = -12.03, *P* < 0.001), this region still harbored majority of these infections especially *A. lumbricoides* and *T. trichiura* species (Table 3).

**Table 3:** Baseline, midterm and endline prevalence (%) of infection and relative reductions % (Wald test: *Z*-statistic, *P*-value) by region among Kenyan school-aged children, 2012–2017

	Baseline	Midterm	Endline	Relative reduction (%) (Baseline – Endline)
	Prevalence % (95% CI)	Prevalence % (95% CI)	Prevalence % (95% CI)	
Coast Region (46 schools)				
STH combined	24.2 (19.9–29.4)	5.4 (3.5–8.4)	3.0 (2.2–4.1)	87.6 (*Z* = -20.24, *P* < 0.001)
Hookworm	18.2 (14.0–23.5)	4.2 (2.5–7.0)	1.6 (1.1–2.3)	91.2 (*Z* = -17.47, *P*<0.001)
*A. lumbricoides*	1.0 (0.7–1.6)	0.3 (0.1–0.5)	0.3 (0.2–0.6)	70.0 (*Z* = -26.00, *P*<0.001)
*T. trichiura*	7.9 (5.7–10.9)	1.6 (1.0–2.7)	1.3 (0.8–2.1)	83.5 (*Z* = -18.93, *P*<0.001)
*S. mansoni*	0 (0–0.1)	0 (0–0.2)	0.1 (0–0.2)	increase
*S. haematobium*	14.8 (11.3–19.5)	6.8 (4.3–10.7)	2.4 (1.3–4.3)	83.8 (*Z* = -17.42, *P* < 0.001)
Nyanza Region (64 schools)				
STH combined	30.6 (27.0–34.6)	15.0 (12.1–18.7)	12.4 (9.8–15.7)	59.4 (*Z* = -20.55, *P* < 0.001)
Hookworm	11.8 (9.8–14.1)	2.4 (1.6–3.6)	1.3 (0.8–2.2)	89.0 (*Z* = -28.36, *P* < 0.001)
*A. lumbricoides*	19.9 (16.0–24.7)	12.6 (9.7–16.2)	10.4 (7.9–13.7)	12.6 (*Z* = -16.16, *P* < 0.001)
*T. trichiura*	3.6 (2.5–5.1)	1.7 (1.2–2.4)	1.6 (1.1–2.4)	55.6 (*Z* = -22.82, *P* < 0.001)
*S. mansoni*	2.8 (1.5–5.0)	0.8 (0.5–1.2)	2.3 (1.2–4.5)	17.9 (*Z* = -11.89, *P* < 0.001)
*S. haematobium*	–	–	–	–
Rift Valley (34 schools)				
STH combined	36.3 (30.4–43.4)	25.7 (20.8–31.8)	26.4 (21.2–32.8)	27.5 (*Z* = -12.03, *P* < 0.001)
Hookworm	3.5 (2.1–6.1)	0.3 (0.2–0.6)	0.4 (0.2–0.9)	88.6 (*Z* = -14.73, *P* < 0.001)
*A. lumbricoides*	27.1 (21.9–33.6)	18.5 (14.6–23.4)	17.5 (13.4–23.1)	35.4 (*Z* = -12.91, *P* < 0.001)
*T. trichiura*	11.9 (7.7–18.4)	11.2 (7.3–17.0)	11.6 (7.8–17.3)	2.5 (*Z* = -10.09, *P* < 0.001)
*S. mansoni*	0.4 (0.1–2.5)	0.4 (01–1.3)	0.5 (0.2–1.2)	increase
*S. haematobium*	–	–	–	–
Western Region (55 schools)				
STH Combined	38.6 (34.9–42.7)	21.2 (17.4–25.9)	15.2 (11.7–19.6)	60.6 (*Z* = -21.46, *P* < 0.001)
Hookworm	24.8 (21.1–29.2)	1.9 (1.3–2.7)	1.6 (1.1–2.2)	93.5 (*Z* = -25.39, *P* < 0.001)
*A. lumbricoides*	24.6 (20.8–29.1)	16.9 (13.8–20.6)	11.1 (8.3–15.2)	54.9 (*Z* = -18.21, *P* < 0.001)
*T. trichiura*	5.9 (3.9–9.0)	6.0 (3.5–10.2)	4.4 (2.6–7.8)	25.4 (*Z* = -12.58, *P* < 0.001)
*S. mansoni*	4.2 (1.9–9.2)	4.2 (1.7–10.5)	3.1 (1.4–6.8)	26.2 (*Z* = -8.29, *P* < 0.001)
*S. haematobium*	–	–	–	–

–, *S. haematobium* was not examined in Nyanza, Rift Valley and Western regions

Whilst the study was powered at a national, not county-level, the results showed some interesting trends in infection heterogeneity by county. Only three counties (Kilifi, Migori and Mombasa) reduced any STH infections by over 90%, while nine and one counties significantly reduced hookworm and *T. trichiura* infections by over 90% respectively. No county reduced *A. lumbricoides* prevalence by over 90%. After five years of MDA, four counties (Kericho, Kisii, Narok and Vihiga) still had prevalence of any STH infections ranging between 20% and < 50%, another four counties (Bomet, Busia, Homa Bay and Nyamira) had prevalence ranging between 10% and < 20%, while seven counties (Bungoma, Kakamega, Kilifi, Kisumu, Kwale, Migori and Mombasa) had their prevalence range between 1% and < 10%, and only Taita Taveta County recorded prevalence below 1% (Table 4). The baseline to endline mean intensity of each STH species and RR by county are presented in Additional file 1: Table S1.

**Table 4:** Baseline, midterm and endline STH prevalence (%) and relative reduction (RR) by county among Kenyan school-aged children, 2012–2017

	STH combined				Hookworm				*A. lumbricoides*				*T. trichiura*			
County	Y1 baseline	Y3 midterm	Y5 endline	RR (%)	Y1 baseline	Y3 midterm	Y5 endline	RR (%)	Y1 baseline	Y3 midterm	Y5 endline	RR (%)	Y1 baseline	Y3 midterm	Y5 endline	RR (%)
Overall	32.3	16.4	13.5	58.2*	15.4	2.3	1.3	91.6*	18.1	11.9	9.6	46.8*	6.7	4.5	4.1	38.4*
Bomet	29.7	23.3	18.1	39.1*	0.2	0.1	0.1	49.9	27.9	20.9	15.2	45.5*	3.9	5.7	4.5	+
Bungoma	49.5	10.9	7.3	85.2*	44.0	1.8	0.9	98.0*	30.7	9.7	6.6	78.6*	0.8	0	0.2	73.5*
Busia	36.1	25.7	16.9	53.3*	20.9	3.1	2.7	87.1*	14.4	15.1	7.2	49.9*	12.5	14.1	10.1	18.9
Homa Bay	30.3	16.4	11.5	62.0*	14.7	5.2	2.7	81.9*	17.3	11.4	7.8	55.2*	5.8	2.9	2.9	50.4*
Kakamega	31.4	15.9	9.8	68.8*	23.1	0.8	0.5	97.7*	23.1	15.0	9.3	59.6*	0.7	0.7	0.3	61.1
Kericho	29.2	16.7	21.0	27.9*	5.7	0.1	0.2	97.2*	24.5	14.6	18.1	26.1*	4.7	4.0	4.8	+
Kilifi	33.5	2.9	2.9	91.3*	28.1	1.4	0.8	97.2*	2.0	0.4	0.5	75.0	6.5	1.4	1.9	70.8*
Kisii	46.8	26.2	23.7	49.4*	11.1	1.4	0.9	92.1*	39.7	25.4	22.8	42.4*	1.3	1.1	1.0	26.9
Kisumu	17.4	4.7	4.0	76.8*	8.4	0.5	0.7	92.2*	7.8	2.4	1.8	77.0*	4.1	2.0	2.0	51.6
Kwale	33.6	11.1	4.8	85.7*	27.7	9.6	3.3	88.1*	0.8	0.4	0.2	75.0	8.9	2.4	1.7	80.9*
Migori	22.3	2.1	2.2	90.2*	20.1	0.7	0.4	98.2*	3.4	1.4	1.7	49.4	0.7	0.1	0.2	65.1
Mombasa	18.1	2.5	1.8	90.1*	5.5	0.8	0.4	92.7*	1.2	0	0.4	66.7*	15.6	1.8	1.0	93.6*
Narok	53.0	39.7	43.1	18.7*	5.0	0.8	1.2	75.0*	29.3	20.3	19.7	32.7*	30.2	26.6	28.6	5.1
Nyamira	31.6	19.1	17.6	44.4*	1.9	0.4	0.1	95.1*	27.6	18.8	17.4	37.0*	3.1	0.5	0.4	87.6*
Taita Taveta	2.5	0.4	0.9	60.0*	0.9	0.1	0.4	55.6*	0.4	0	0.3	25.0	1.4	0.3	0.3	78.6*
Vihiga	50.2	35.9	32.8	34.7*	16.0	1.8	2.2	86.1*	44.4	33.9	30.0	32.5*	9.9	7.2	6.7	32.2

*Statistically significant (*P* < 0.05) relative reduction in prevalence

+ indicates an increase rather than relative reduction in prevalence

**Fig. 3:**
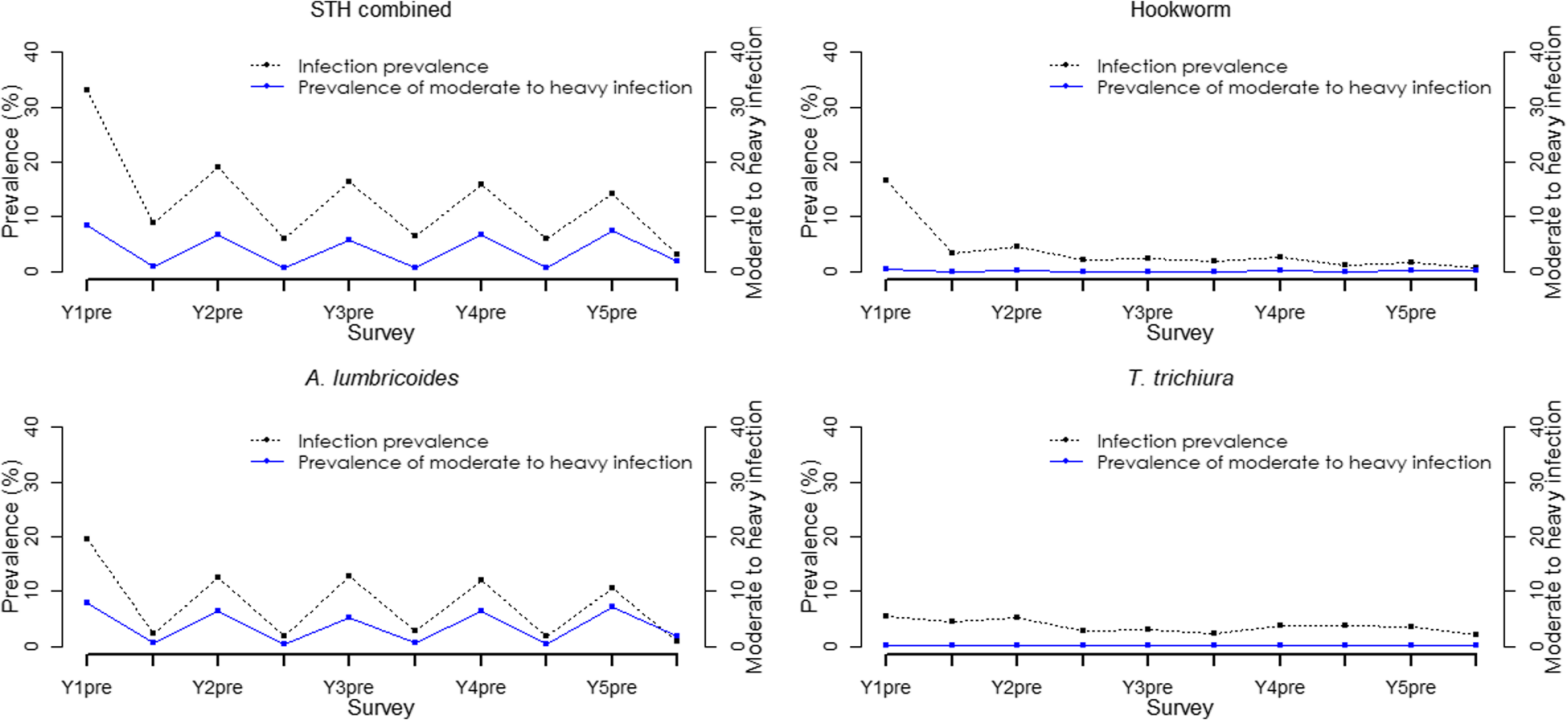
Trend in STH prevalence among Kenyan school-aged children, 2012–2017

**Fig. 4:**
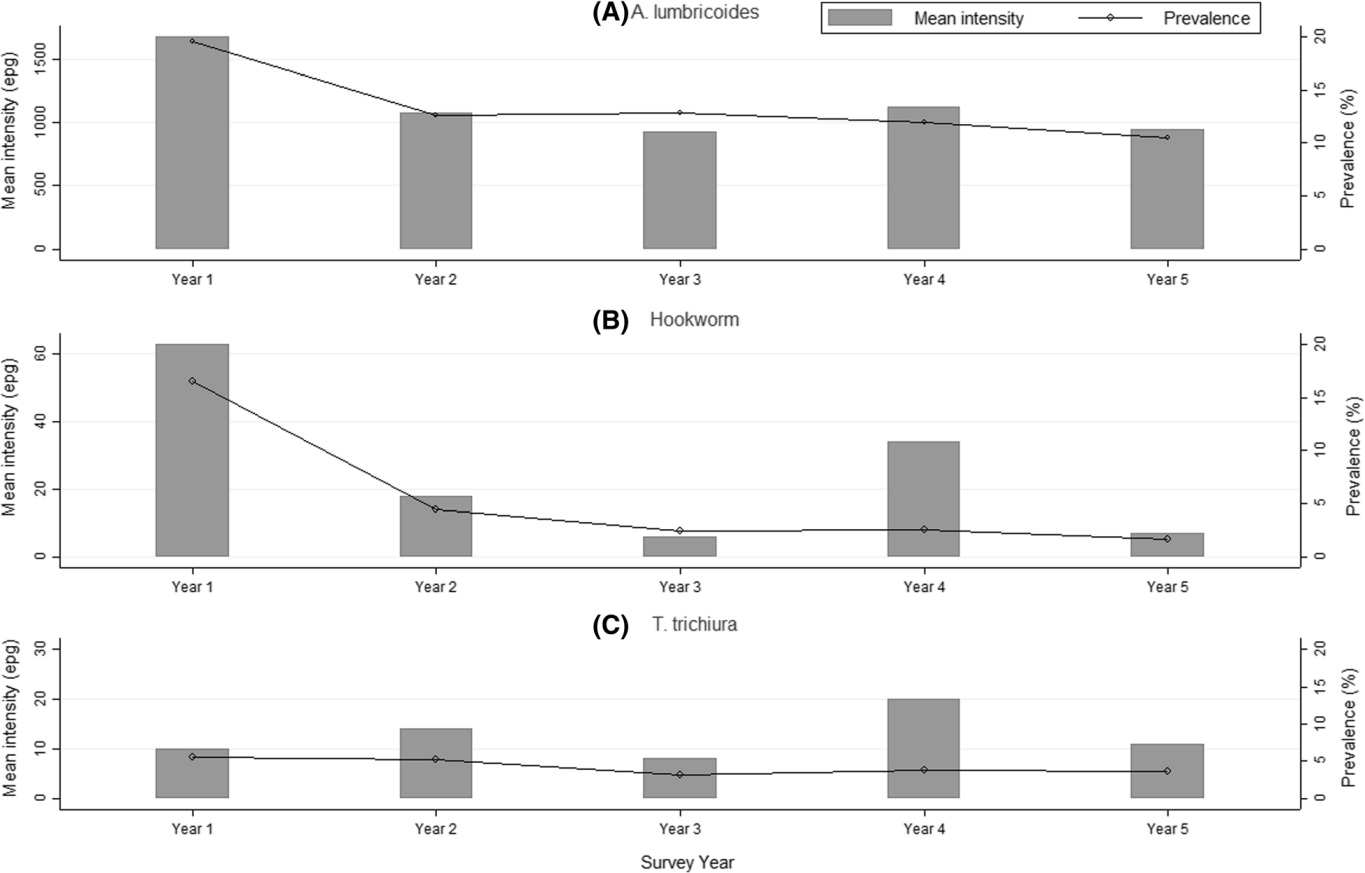
Mean intensity and prevalence of STH infections among Kenyan school-aged children

The trend in STH prevalence since baseline was assessed based on the 59 schools category. The overall prevalence of each specific species and STH combined has declined over the five years to below 3% as outlined in Fig. 3. Further, relationship between mean intensity and prevalence for STH infections is shown in Fig. 4.

Among the 59 schools, the prevalence of light, moderate and heavy infections substantially reduced between baseline and endline. The overall relative reductions were as follows: for STH combined (light: 90.1%, moderate: 94.0%, heavy: *increased*), for hookworm (light: 88.2%, moderate: 82.6%, heavy: 79.1%), for *A. lumbricoides* (light: 97.0%, moderate: 95.3%) and *T. trichiura* (light: 39.9%). We did not observe reductions in moderate and heavy infections for *T. trichiura*. Similarly, we noted a general declining trend in the prevalence of moderate to heavy intensity of infections from baseline to endline. At baseline, the STH combined prevalence of moderate to heavy infections was 8.4% (95% CI: 6.3*–*11.2%) and after five rounds of MDA at year 5 post-MDA it reduced to 1.9% (95% CI: 1.5*–*2.5%) translating to a significant relative reduction of 77.1% (Wald test: *Z* = -7.96, *P* < 0.001). The trend in prevalence of moderate to heavy intensity of STH infections is shown in Fig. 3.

In terms of STH re-infection patterns assessed in the 59 schools, we found that after one year of MDA delivery, the re-infection in prevalence for STH combined was 14.0% with *A. lumbricoides* showing the highest re-infection levels of 7.5%, followed by *T. trichiura* at 4.8% and hookworm at 3.9%. However, after five years of MDA delivery, the fifth year re-infection levels did not reduce significantly, with the STH combined re-infection in prevalence being 10.4% (6.7% for *A. lumbricoides*; 3.7% for *T. trichiura*; and 1.4% for hookworm). A similar re-infection pattern was seen for the mean intensity of STH infections.

### Schistosome infections

**Fig. 5:**
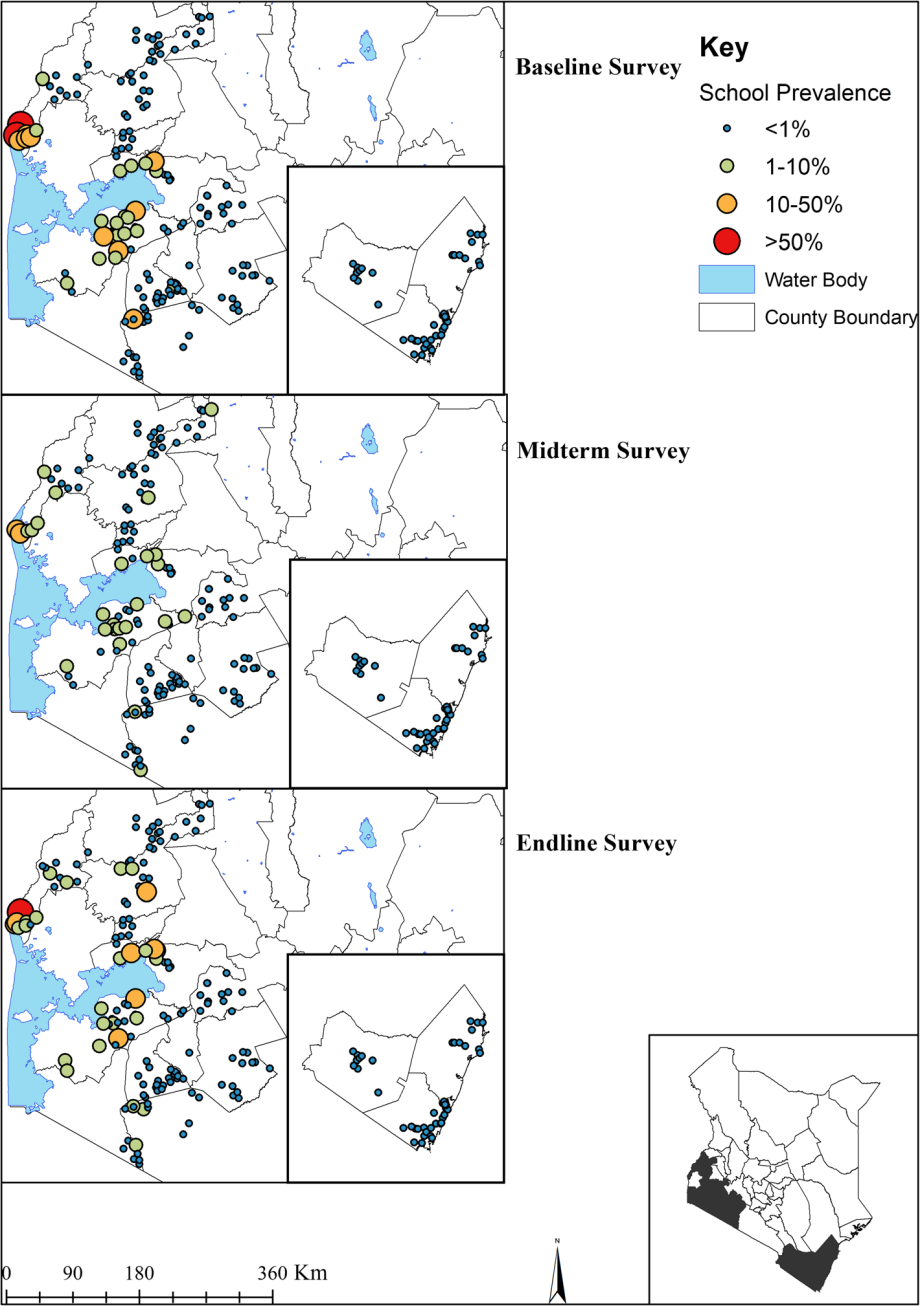
The geographical distribution of *S. mansoni* infection prevalence at baseline (2012), midterm (2015) and endline (2017) among Kenyan school-aged children

**Fig. 6:**
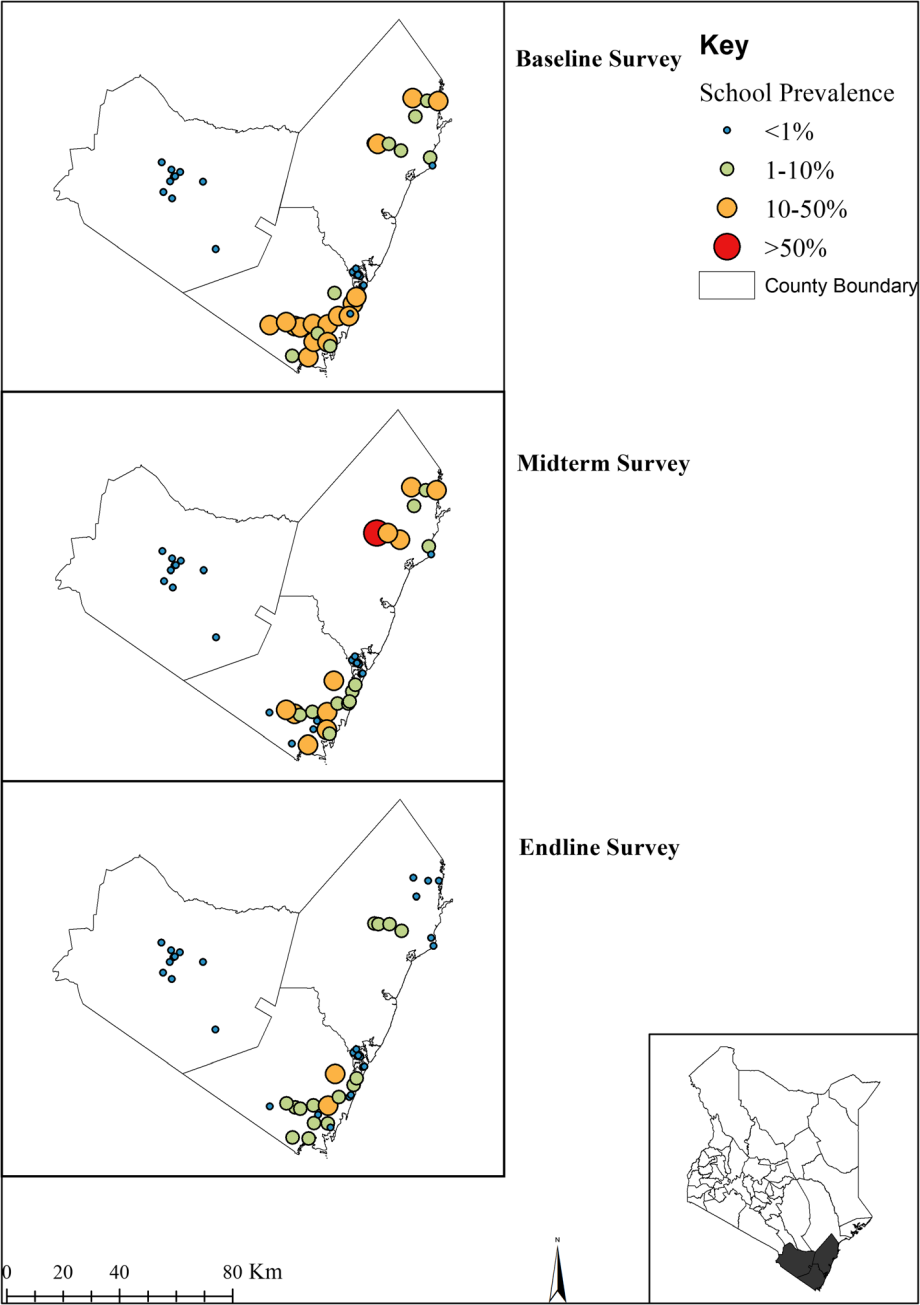
The geographical distribution of *S. haematobium* infections prevalence at baseline (2012), midterm (2015) and endline (2017) among Kenyan school-aged children

Figures 5 and 6 provide the geographical distribution of *S. mansoni* and *S. haematobium* infections prevalence from baseline to endline. The overall prevalence of *S. mansoni* was 2.1% (95% CI: 1.3–3.5%), 1.5% (95% CI: 0.7–3.1%) and 1.7% (95% CI: 1.0–2.8%) for baseline, midterm and endline surveys, respectively, with respective mean infection intensities of 12 epg (95% CI: 4–36), 5 epg (95% CI: 2–14) and 5 epg (95% CI: 2–9). In the Coast Region where urine samples were collected, the overall prevalence of *S. haematobium* was 14.8% (95% CI: 11.3–19.5%), 6.8% (95% CI: 4.3–10.7%) and 2.4% (95% CI: 1.3–4.3%) for baseline, midterm, and endline surveys, respectively, with respective mean infection intensities of 16 epg (95% CI: 10–26), 7 epg (95% CI: 4–12), and 2 epg (95% CI: 1–5). After five years, the mean intensity of *S. mansoni* significantly reduced by 61.6% (Wald test: *Z* = -3.10, *P* = 0.002); however, its prevalence reduction was not significant (Wald test: *Z* = -1.86, *P* = 0.062). Both the prevalence and mean intensity of *S. haematobium* significantly reduced by 84.0% (Wald test: *Z* = -5.74, *P* < 0.001) and 87.2% (Wald test: *Z* = -4.18, *P* < 0.001) respectively (Table 2).

*Schistosoma mansoni* infection prevalence and mean intensity significantly varied by regions at all the three national survey points and its prevalence was still highest in Western and Nyanza regions at 3.1% and 2.3%, respectively, during the endline survey (Table 3).

Reductions in schistosome infections were heterogeneous by county. Ten counties showed an increase instead of relative reduction in *S. mansoni* prevalence with no county significantly reducing *S. mansoni* prevalence by even 50% over the five-year period. At endline survey, most *S. mansoni* infections were seen in three counties [Busia (8.0%), Kisumu (5.4%) and Homabay (3.7%)] while the rest of the counties showed prevalence of below 1% except for Narok County (1.3%). *Schistosoma haematobium* was only evaluated in four counties with Kwale and Kilifi counties most prevalent for *S. haematobium* at 5.3% and 1.5%, respectively, after five years, and the remaining two counties (Mombasa and Taita Taveta) maintaining no infection level over the five-year period (Table 5). Baseline to endline mean intensity of each schistosome infection species and RR by county are presented in Additional file 1: Table S2.

**Table 5:** Baseline, midterm and endline schistosomiasis prevalence (%) and relative reduction (RR) by county among Kenyan school-aged children, 2012–2017

	*S. mansoni*				*S. haematobium*			
County	Y1 baseline	Y3 midterm	Y5 endline	RR (%)	Y1 baseline	Y3 midterm	Y5 endline	RR (%)
Overall	2.1	1.5	1.7	19.3	14.8	6.8	2.4	84.0*
Bomet	0	0.2	0.3	+	–	–	–	–
Bungoma	0	0.1	0.1	+	–	–	–	–
Busia	12.6	12.1	8.0	36.4*	–	–	–	–
Homa Bay	5.8	1.7	3.7	37.4*	–	–	–	–
Kakamega	0.1	0.3	1.0	+	–	–	–	–
Kericho	0	0.2	0	0	–	–	–	–
Kilifi	0	0	0.1	+	10.0	12.6	1.5	85.0*
Kisii	0.2	0	0.3	+	–	–	–	–
Kisumu	3.1	1.0	5.4	+	–	–	–	–
Kwale	0.1	0.1	0	100	17.5	8.4	5.3	69.7*
Migori	0	0.3	0.4	+	–	–	–	–
Mombasa	0	0.1	0.3	+	0	0	0	0
Narok	1.2	1.0	1.3	+	–	–	–	–
Nyamira	0.4	0	0.1	74.4	–	–	–	–
Taita Taveta	0	0	0	0	0	0	0	0
Vihiga	0	0.1	0.4	+	–	–	–	–

*Statistically significant (*P* < 0.05) relative reduction in prevalence

+ indicates an increase rather than relative reduction in prevalence

– indicates areas where survey for *S. haematobium* was not undertaken

**Fig. 7:**
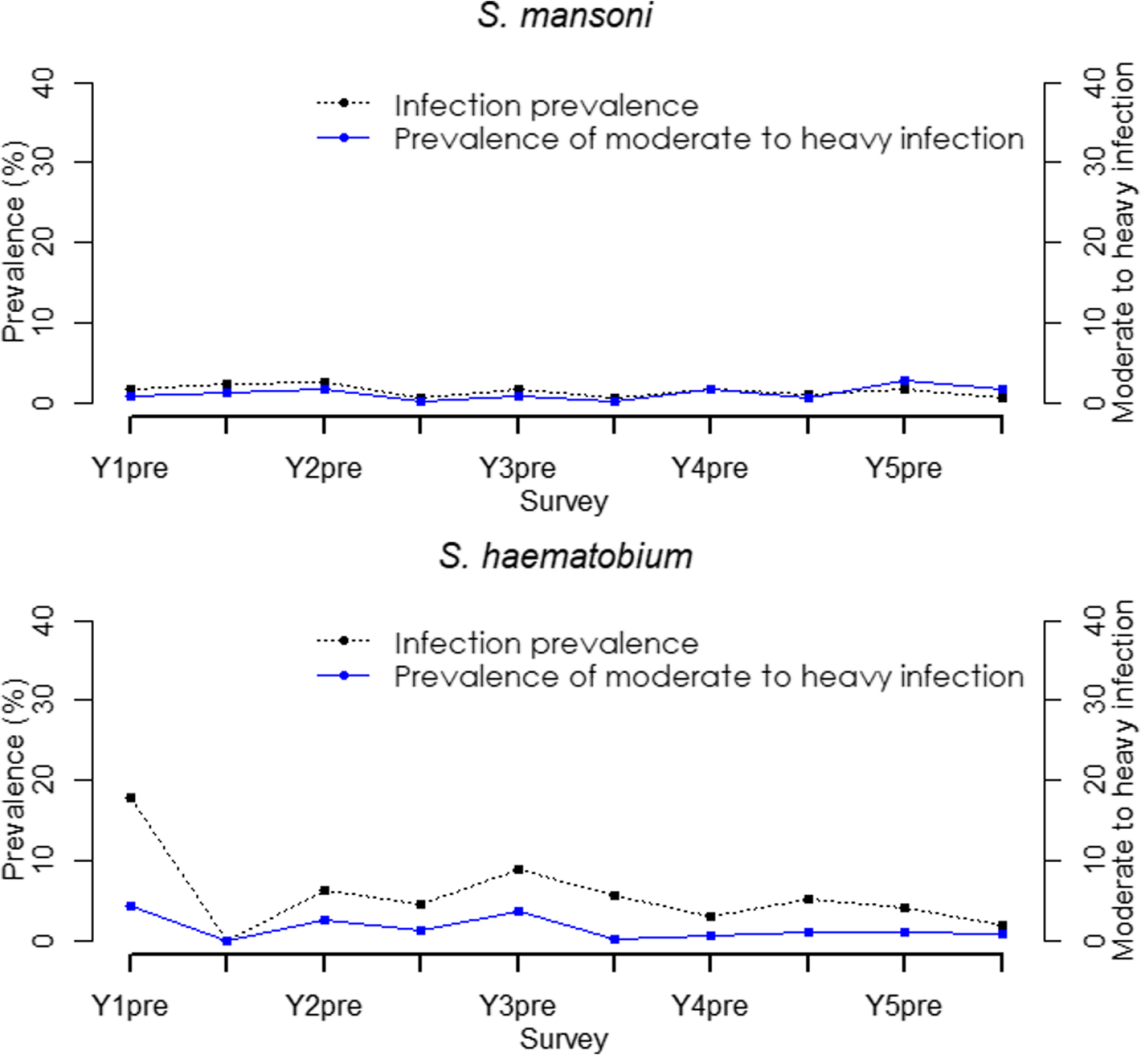
Trend in schistosome infections prevalence among Kenyan school-aged children, 2012–2017

The trend in schistosome infections over the five years was based on the 59 schools category, where we noted that the prevalence of any schistosome infections had significantly reduced by 59.3% (Wald test: *Z* = -3.03, *P* = 0.002) while for specific species prevalence, only *S. haematobium* showed significant reduction of 77.1% (Wald test: *Z* = -2.59, *P* = 0.010). The immediate pre- and post-MDA significant reductions in prevalence for any schistosome infections was seen only in year 2 (RR = 56.0%, Wald test: *Z* = -3.24, *P* = 0.001), year 3 (RR = 42.9%, Wald test: *Z* = -2.02, *P* = 0.044) and year 5 (RR = 56.1%, Wald test: *Z* = -2.78, *P* = 0.005) but not in year 1 and 4. Fig. 7 provides the trend in schistosome infections prevalence from year 1 to 5.

The analysis of the prevalence of light, moderate and heavy intensity of schistosome infections showed an increase in prevalence of both light and heavy intensity of infections for *S. mansoni* from baseline to endline with only moderate intensity of infection showing a non-significant decrease of 10.8% (Wald test: *Z* = -0.60, *P* = 0.548). For *S. haematobium*, the overall prevalence of both light and heavy intensity reduced significantly by 11.1% (Wald test: *Z* = -2.45, *P* = 0.014) and non-significantly by 73.4% (Wald test: *Z* = -1.62, *P* = 0.105) respectively. Fig. 7 shows the trend in prevalence of moderate to heavy intensity of schistosome infections.

Re-infection in prevalence for any schistosome infections has significantly remained high since baseline, the re-infection rates for any schistosome infections were; 21.9%, 10.8%, 8.9% and 9.8% after year 1, 2, 3 and 4 MDA deliveries, respectively, while the re-infection rates for *S. haematobium* has for the last four MDAs been high (between 4–7%) compared to those for *S. mansoni* (which were between 1–3%).

### Comparison of both STH and schistosome infections prevalence among early childhood (ECD) and older children

According to the design of the M&E programme, six classes (i.e. one ECD class and classes 2–6) in each survey school was targeted for sample collection. Comparison of infection prevalence among ECD and older children revealed that ECD children are more likely to be infected with STH infections, across all the survey years, compared to the older children (OR = 1.22, *P* < 0.001) but less likely to be infected with any schistosome infections (OR = 0.81, *P* = 0.003). The comparison of prevalence for both STH and schistosome infections among ECD and older children is outlined in Table 6.

**Table 6:** Comparison of the overall infection prevalence among ECD and older children, 2012–2017

Year/Survey	No. sampled (%)	STH combined prevalence (95% CI)	Any schistosome prevalence (95% CI)	Prevalence of STH combined moderate to heavy intensity (95% CI)	Prevalence of any schistosome moderate to heavy intensity (95% CI)
Year 1: Baseline					
ECD children	0	0	0	0	0
Older children	3193 (100)	32.7 (29.2–36.5)	25.9 (16.4–40.8)	6.2 (4.5–8.6)	2.8 (1.3–6.4)
Year 1: Post-MDA					
ECD children	903 (15.7)	9.0 (6.3–12.8)	*	1.1 (0.6–2.2)	1.1 (0.4–2.9)
Older children	4865 (84.3)	9.1 (6.8–12.1)	*	0.8 (0.5–1.3)	1.5 (0.7–3.4)
Year 2: Pre-MDA					
ECD children	267 (16.5)	10.9 (5.1–23.2)	3.7 (1.4–10.4)	1.1 (0.4–3.2)	1.5 (0.6–3.6)
Older children	1347 (83.5)	11.4 (6.1–21.2)	6.9 (3.6–13.3)	0.4 (0.2–1.3)	2.8 (1.4–5.7)
Year 2: Post-MDA					
ECD children	–	–	–	–	–
Older children	–	–	–	–	–
Year 3: Midterm					
ECD children	3439 (16.3)	19.9 (17.4–22.7)	13.8 (9.0–21.1)	8.6 (7.1–10.4)	1.3 (0.7–2.6)
Older children	17624 (83.7)	15.6 (13.7–17.9)	12.9 (8.4–19.9)	5.2 (4.3–6.2)	1.3 (0.7–2.4)
Year 3: Post-MDA					
ECD children	1026 (16.7)	7.6 (5.3–10.9)	7.5 (3.7–15.1)	1.6 (0.9–2.8)	0.2 (0–0.8)
Older children	5136 (83.3)	6.1 (4.5–8.3)	8.8 (4.9–15.6)	0.6 (0.3–0.9)	0.3 (0.2–0.5)
Year 4: Pre-MDA					
ECD children	1011 (16.3)	21.2 (16.3–27.4)	7.8 (3.1–19.7)	10.4 (7.6–14.2)	2.2 (1.0–4.6)
Older children	5183 (83.7)	15.0 (11.9–18.9)	9.9 (3.7–26.3)	6.2 (4.6–8.4)	2.0 (0.9–4.4)
Year 4: Post-MDA					
ECD children	824 (16.6)	6.8 (4.5–10.2)	6.8 (3.1–14.7)	0.6 (0.2–1.7)	0.4 (0.1–1.1)
Older children	4143 (83.4)	5.6 (4.0–7.8)	9.0 (4.2–19.4)	0.7 (0.3–1.3)	1.0 (0.4–2.5)
Year 5: Endline					
ECD children	3424 (16.5)	17.3 (14.8–20.2)	7.8 (4.4–14.1)	10.5 (8.8–12.4)	2.8 (1.9–4.2)
Older children	17386 (83.6)	12.7 (10.9–14.9)	9.5 (6.2–14.6)	6.0 (5.0–7.2)	2.2 (1.6–3.0)
Year 5: Post-MDA					
ECD children	1033 (16.7)	5.1 (3.5–7.4)	6.4 (2.9–14.1)	3.2 (2.1–4.8)	2.9 (1.8–4.7)
Older children	5147 (83.3)	2.6 (1.5–4.3)	4.5 (2.3–8.8)	1.7 (1.3–2.3)	1.8 (1.3–2.5)

*Key*: *, insufficient observation; –, child demographic details were not collected during year 2 post-MDA

### Treatment coverage

Annual deworming for STH infections using albendazole was carried out for five years in 28 counties and all the 16 counties included in the M&E programme were covered for treatment as per the WHO guidelines. Since baseline in 2012, a total of 5.9, 6.4, 6.1, 6.4 and 5.9 million children were dewormed for STH in year 1, 2, 3, 4 and 5 in all the 28 counties, with overall treatment coverage of 81.3%, 77.5%, 83.0%, 80.0% and 76.3% respectively.

**Fig. 8:**
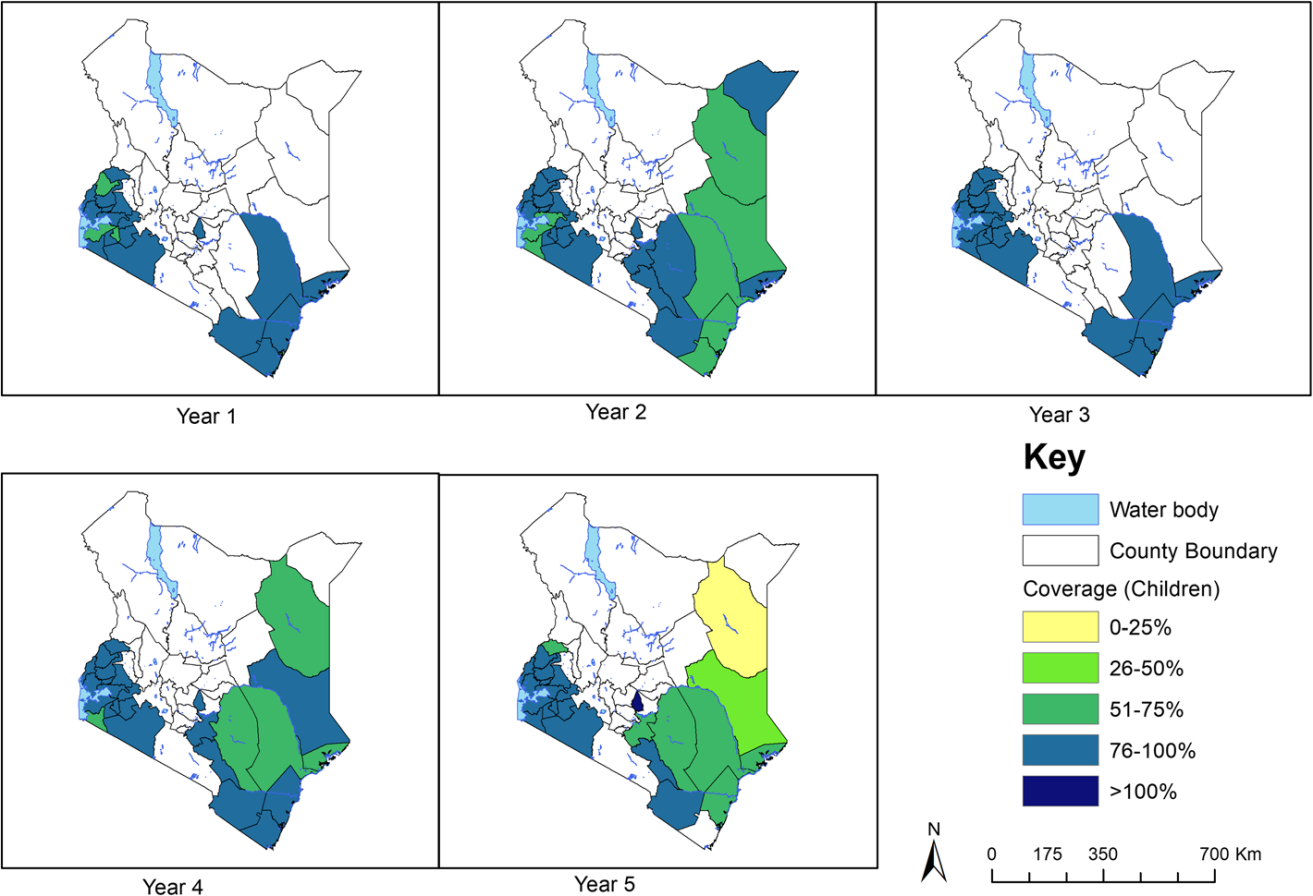
STH treatment coverage (children), 2012–2017

**Fig. 9:**
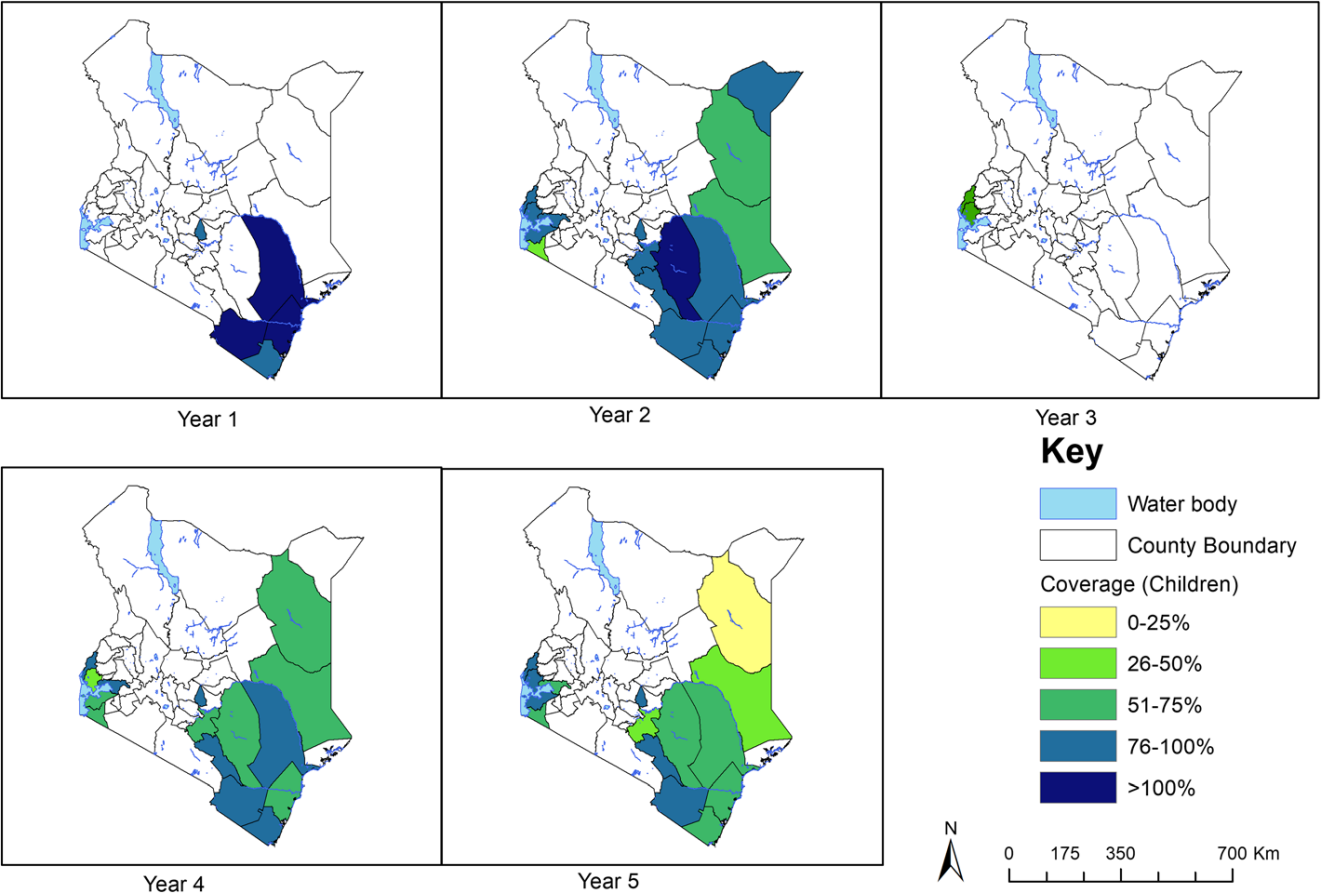
Schistosome treatment coverage (children), 2012–2017

On the other hand, the annual deworming for schistosome infections using praziquantel was not consistently carried out, and covered fewer counties especially those monitored by the M&E programme. In year 1, only 5 out of the 28 counties received praziquantel with approximately 191,318 children (average coverage of 104%) being dewormed; in year 2, only 16 out of the 28 counties received treatment with approximately 890,459 children (average coverage of 84.7%) being dewormed; in year 3, only 2 of 28 counties received treatment reaching only 79,038 children (average coverage of 81.7%); in year 4 and 5, 15 of 28 counties were treated for each of the two years for schistosome infections covering approximately 556,638 and 519,232 children with average coverage of 73.4% and 65.6% respectively. Treatment coverage for STH and schistosome infections by county is shown in Figs. 8 and 9 respectively.

## Discussion

This study provides a rigorous assessment of both STH and schistosome infections following a 5-year national school-based deworming programme. Data revealed considerable declines in the prevalence and mean intensity of both infections from baseline to endline surveys as well as significant yearly reductions in the infections, an indication that the programme has greatly reduced the infections. Importantly however, in most places, these declines were not to a level where the infections are no longer a public health concern or will lead to attainment of the transmission breaking point. As such, alternative and or additional strategies alongside the school-based preventive chemotherapy, expansion of other at-risk cohorts, and enhanced diagnostic tools will be needed to achieve elimination of these infections as a public health problem.

### STH infections

The most common STH infection detected among the Kenyan children during baseline survey was *A. lumbricoides*, followed by hookworm and *T. trichiura* respectively. After five years of MDA, *T. trichiura* infection was the second most diagnosed infection after *A. lumbricoides*. Hookworm infection showed the highest relative reduction in both its prevalence and intensity followed by *A. lumbricoides*. This indicates that the annual single-dose oral albendazole given to the school children by the programme is efficacious against hookworm and *A. lumbricoides* infections but not *T. trichiura*, a finding supported by previous studies [20, 21].

According to regions, Coast Region showed the highest reduction in any STH infections followed by Western and Nyanza regions with Rift Valley still harboring majority of the STH infections. The greatest reduction of STH infections in the Coast can partly be attributed to the other additional interventions in the region; the two-year community-wide cluster randomized trial by the TUMIKIA project that provided albendazole to 120 community clusters in Kwale County [22] and the National Programme for Elimination of Lymphatic Filariasis (NPELF) in the wider Coast Region which had distributed over five rounds of diethylcarbamazine citrate (DEC) and albendazole [23].

According to counties, heterogeneity in STH infections was observed with only three counties reducing any STH infections by over 90%, and with varied county reduction in prevalence and mean intensity of species-specific infections. The county level heterogeneity can be attributed to the specific epidemiological situation, geographical diversity, initial prevalence levels or worm burden, different transmission intensities, as well as differing treatment coverage, drug uptake and compliance [8, 14].

The trends and patterns of STH infections as assessed by pre- and post-intervention surveys showed that the overall and species-specific infections had declined over the five years period. This substantial decline may be attributed to the direct impact of the programme and any other school health interventions.

The five-year results showed a general decline in the prevalence of light, moderate and heavy intensity of STH infections, as defined by WHO. The overall reduction of moderate to heavy intensity of any STH infections reduced by over 77% while that of *A. lumbricoides* reduced by similar percentage and hookworm reduced by over 81%. *Trichuris trichiura* infection did not record any reduction in its moderate to heavy infection intensity. This shows that the programme managed to substantially reduce the morbidity associated with STH infections especially hookworms and *A. lumbricoides*, using preventive chemotherapy alone but that gain could be reversed by the higher re-infection levels and lack of additional interventions in areas with high transmission rates.

We recorded higher re-infection rates particularly for *A. lumbricoides* compared to other species. This finding is in line with that of Jia et al. [24] who in their systematic review of STH re-infection after treatment, evaluated 24 studies and observed that re-infections occur rapidly after treatment particularly for *A. lumbricoides* and *T. trichiura*. Moreover, Zerdo et al. [25] evaluated STH re-infection among school-aged children in southern Ethiopia and noted similar results. Whilst it is clear that each MDA round was effective in reducing STH prevalence and intensity, the re-infection rate remained problematic. Hence, there is need for additional control approaches emphasizing health education and WASH in order to maximize the benefit of preventive chemotherapy.

STH parasite infections were more prevalent in younger (ECD) children than the older ones, underscoring the importance of delivery of deworming drugs to pre-school-aged children. Their possible frequent interaction with contaminated human faeces, swallowing contaminated water or walking barefoot in contaminated soil could have enhanced their risk. This points to a higher level of infection among the non-enrolled children who are in the community. This high prevalence among ECD children might be explained by the low MDA coverage among this cohort of children. This group remains an important source of on-going transmission and there is need to invest in innovative ways of reaching pre-school and non-enrolled children [26]. To accelerate the attainment of WHO target of eliminating STH infections by the year 2020 [5], the NSBDP might need to increase its treatment coverage of the pre-school and non-enrolled children population.

### Schistosome infections

Treatment delivery for both schistosome infections was inconsistent with some counties going without treatment in some years. The challenge notwithstanding, the programme reduced infections associated with schistosomiasis from initial level of 2.1% to 1.7% for *S. mansoni* and 14.8% to 2.4% for *S. haematobium*. A recent systematic review by Lai et al. [27] among studies done in 44 countries of sub-Saharan Africa targeting school-aged children revealed a similar estimate of 18.5% for *S. haematobium* in the year 2012, and the authors noted that infection risk had continued to decrease gradually.

Prevalence and intensity of schistosome infections varied markedly by regions and counties with more infections observed in Western and Nyanza regions. After five years of the programme implementation, three counties (Busia, Kisumu and Homa Bay) had higher *S. mansoni* infections and two counties (Kilifi and Kwale) had higher *S. haematobium* infections. Evidence suggests that *Schistosoma* infection and associated disease exhibit important micro-geographical heterogeneities with divergent patterns for *S. mansoni* and *S. haematobium* with observed extreme focality even at community level [28, 29]. Accordingly, current strategies need to consider local geospatial patterns in order to move from morbidity control to elimination of schistosomiasis.

Re-infection rates for schistosome infections has been relatively high especially for *S. haematobium* compared to those for STH infections. This could be due to the fact that praziquantel is highly effective in killing adult schistosome worms, but not juvenile schistosomes, therefore unable to prevent re-infection [30]. Thus, a control programme based solely on provision of praziquantel is not effective or sustainable in the long term. Development of a multi-faceted, integrated control programme would have a greater and longer lasting effect on disease transmission than the current chemotherapy-based programmes [31]. Molluscicides have shown to be effective for the control of the intermediate host (snail) of schistosomiasis and this appears to be environmentally acceptable alternative. Where possible, control of the schistosome vector snail will greatly decrease the presence of the parasites in transmission sites, thus, reducing the infections in the communities [32].

As much as treatment delivery for schistosomiasis using praziquantel was inconsistent in some parts of the country, the results showed that MDA intervention with praziquantel has major benefits for the infected children by reducing any schistosome infections by 64.5%. However, MDA by itself has done little to reduce schistosome re-infections and heavy intensity of infections. Recently, other authors [30, 33, 34] have suggested snail control in endemic areas as a key contemporary measure to reducing schistosomiasis and even accelerating the attainment of WHO 2020 target of eliminating schistosomiasis.

### Limitations

Major limitations of this study are: (i) The choice of diagnostic tool; the study used Kato-Katz technique which is the WHO recommended tool and previously shown as user-friendly, robust and accurate for stool examination of STH and *S. mansoni* eggs in high endemic settings [35]. However, it has been shown as less sensitive in areas of low prevalence and intensity of infection [17], therefore our prevalence estimates are likely to be under-estimates of the true population prevalence, especially at endline where intensity of infection was driven down through five years of MDA. (ii) The school sampling method; the study used similar sampling criteria for both STH and schistosome infections; however, this seemed to have under-estimated schistosome infections since they are largely focal and perhaps more schools ought to have been sampled in a particular county to get an accurate estimate of the schistosome infections. (iii) The study was statistically powered to detect changes in infection prevalence at the national and regional level only, hence the estimates at the county level may have been under-estimated. Lastly, (iv) the study only followed schools and not individual children longitudinally. Whilst this was appropriate for programmatic evaluation, this presented some fluctuations in the number of individuals with moderate to heavy intensity of infections surveyed across the years. Additionally, it posed challenge in estimating re-infections, which we eventually measured as an increase in infection levels by schools though this may be influenced by variations in individual infections.

## Conclusions

This study showed that enhanced and effective national deworming programmes can attenuate the burden of STH and schistosome infections among the school-aged children; however, complimentary efforts like provision of safe water, sanitation and hygiene as well as escalation of snail control programmes are inevitable if Kenya is to overcome the war against NTDs. It is expected that MDA with school-based deworming alone cannot break the infection transmission cycle and the above complimentary interventions will have to be put in place. Importantly, under investigation is the possibility of interrupting STH transmission by community-wide, biannual MDA [22]. The schistosome infection transmission requires the snail, humans, parasites and suitable habitats like water. As such, reduction of risk factors like presence of snails (intermediate host) is essential for the control of schistosomiasis, since snails are an essential link to the proliferation of the parasites. Therefore, a more concerted and targeted approach will be even more relevant in view of World Health Assembly (WHA) resolution on the elimination of STH (WHA66.20) [36] and schistosomiasis (WHA65.21) [37]. The analysis of these data collected over five-year period provided a robust assessment of the national programme and outlined the current prevalence, mean intensity and re-infection pattern of these infections in Kenya. Our findings will allow the Government of Kenya to make informed decisions on the breaking transmission strategy for these NTDs and prudent allocation of resources towards the adoption of those strategies.

## Additional file

Additional file 1: **Figure S1.** Outline of the 5-year M&E programme. **Table S1.** Baseline, midterm and endline STH mean intensity (epg) and relative reduction (RR) among Kenyan school children, 2012–2017. **Table S2.** Baseline, midterm and endline schistosomiasis mean intensity (epg) and relative reduction (RR) among Kenyan school children, 2012–2017. (DOCX 44 kb)

## Abbreviations

CI: Confidence interval
DEC: Diethylcarbamazine citrate
ECD: Early childhood education
epg: Eggs per gram
KEMRI: Kenya Medical Research Institute
M&E: Monitoring and evaluation
MDA: Mass drug administration
MoE: Ministry of Education
MoH: Ministry of Health
NPELF: National Programme for Elimination of Lymphatic Filariasis
NSBDP: National school-based deworming programme
NTD: Neglected tropical diseases
ODK: Open data kit
RR: Relative reduction
SBD: School-based deworming
STH: Soil-transmitted helminths
WASH: Water sanitation and hygiene
WHA: World Health Assembly
WHO: World Health Organization
