# Supplementary material for: Results of a national school-based deworming programme on soil-transmitted helminths infections and schistosomiasis in Kenya: 2012–2017
Source: Parasit Vectors. 2019 Feb 7;12:76. doi: 10.1186/s13071-019-3322-1 (PMC6367841; doi:10.1186/s13071-019-3322-1)
Supplement: Supplementary file 1 — Figure S1. Outline of the 5-year M&E programme. Table S1. Baseline, midterm and endline STH mean intensity (epg) and relative reduction (RR) among Kenyan school children, 2012–2017. Table S2. Baseline, midterm and endline schistosomiasis mean intensity (epg) and relative reduction (RR) among Kenyan school children, 2012–2017. (DOCX 44 kb) [file 13071_2019_3322_MOESM1_ESM.docx]

**Additional file 1**

**Figure S1: Outline of the 5-year M&E programme**

**Year 2 (2013-14)**

**Year 1 (2012-13)**

**Year 3 (2014-15)**

**Year 5 (2017)**

**Year 4 (2016)**

MDA

60 pre-post schools

60 pre-post schools

MDA

60 pre-post schools

60 pre-post schools

MDA

200 schools (including 60 pre-post schools)

60 pre-post schools

MDA

200 baseline schools (including 60 pre-post schools)

60 pre-post schools

MDA

200 schools (including 60 pre-post schools)

60 pre-post schools

**Table S1: Baseline, midterm and endline STH mean intensity (epg) and relative reduction (RR) among Kenyan school children, 2012 – 2017**

|  | **Hookworm** | | | | ***A. lumbricoides*** | | | | ***T. trichiura*** | | | |
| --- | --- | --- | --- | --- | --- | --- | --- | --- | --- | --- | --- | --- |
| **County** | **Y1  baseline** | **Y3**  **midterm** | **Y5  endline** | **RR (%)** | **Y1  baseline** | **Y3**  **midterm** | **Y5  endline** | **RR (%)** | **Y1  baseline** | **Y3**  **midterm** | **Y5  endline** | **RR (%)** |
| *Overall* | *63* | *8* | *10* | *84.2** | *1659* | *960* | *917* | *44.7** | *33* | *17* | *16* | *50.9* |
| Bomet | 0 | 0 | 0 | 49.9* | 3840 | 1488 | 1262 | 67.1* | 6 | 17 | 7 | + |
| Bungoma | 270 | 1 | 1 | 99.7* | 1566 | 813 | 786 | 49.8* | 10 | 0 | 0 | 97.2* |
| Busia | 112 | 6 | 6 | 94.9* | 878 | 1284 | 634 | 27.6 | 33 | 59 | 25 | 24.9 |
| Homa Bay | 27 | 30 | 8 | 69.5* | 1001 | 798 | 480 | 52.1* | 5 | 9 | 11 | + |
| Kakamega | 129 | 1 | 26 | 79.6 | 1425 | 1156 | 1112 | 22.0 | 1 | 1 | 3 | + |
| Kericho | 14 | 0 | 0 | 99.4* | 2738 | 1232 | 1494 | 45.4* | 18 | 11 | 20 | + |
| Kilifi | 47 | 3 | 0 | 100* | 28 | 4 | 14 | 50.0* | 6 | 3 | 3 | 50.0 |
| Kisii | 23 | 11 | 49 | + | 5147 | 2180 | 2591 | 49.7* | 1 | 1 | 4 | + |
| Kisumu | 15 | 0 | 0 | 96.9* | 423 | 250 | 188 | 55.5 | 11 | 6 | 3 | 71.2 |
| Kwale | 117 | 28 | 10 | 91.5* | 15 | 35 | 6 | 60.0* | 15 | 6 | 7 | 53.3* |
| Migori | 19 | 1 | 0 | 98.5* | 131 | 38 | 98 | 25.4 | 6 | 0 | 0 | 100 |
| Mombasa | 45 | 1 | 0 | 100* | 70 | 0 | 6 | 91.4* | 17 | 1 | 1 | 94.1* |
| Narok | 44 | 2 | 19 | 56.8* | 3822 | 1539 | 1786 | 53.3* | 78 | 134 | 159 | + |
| Nyamira | 1 | 0 | 0 | 99.0* | 3031 | 1523 | 1829 | 39.7 | 385 | 3 | 0 | 99.9* |
| Taita Taveta | 1 | 0 | 4 | + | 21 | 0 | 32 | + | 1 | 0 | 6 | + |
| Vihiga | 103 | 11 | 19 | 81.8* | 3981 | 3191 | 3100 | 22.1 | 31 | 13 | 25 | 18.0 |
| *indicates a statistically significant (p<0.05) relative reduction in mean intensity  +indicates an increase rather than relative reduction in mean intensity | | | | | | | | | | | | |

**Table S2: Baseline, midterm and endline schistosomiasis mean intensity (epg) and relative reduction (RR) among Kenyan school children, 2012 – 2017**

|  | ***S. mansoni*** | | | | ***S. haematobium*** | | | |
| --- | --- | --- | --- | --- | --- | --- | --- | --- |
| **County** | **Y1  baseline** | **Y3**  **midterm** | **Y5  endline** | **RR (%)** | **Y1  baseline** | **Y3**  **midterm** | **Y5  endline** | **RR (%)** |
| *Overall* | *12* | *5* | *5* | *61.6** | *16* | *7* | *2* | *87.2** |
| Bomet | 0 | 7 | 0 | 0 | - | - | - | - |
| Bungoma | 0 | 0 | 0 | 0 | - | - | - | - |
| Busia | 123 | 49 | 28 | 77.0* | - | - | - | - |
| Homa Bay | 6 | 2 | 5 | 21.1 | - | - | - | - |
| Kakamega | 0 | 0 | 7 | + | - | - | - | - |
| Kericho | 0 | 0 | 0 | 0 | - | - | - | - |
| Kilifi | 0 | 0 | 1 | + | 9 | 11 | 0 | 99.5* |
| Kisii | 0 | 0 | 2 | + | - | - | - | - |
| Kisumu | 3 | 1 | 11 | + | - | - | - | - |
| Kwale | 0 | 0 | 0 | 0 | 20 | 9 | 5 | 75.0 |
| Migori | 0 | 0 | 0 | 0 | - | - | - | - |
| Mombasa | 0 | 0 | 1 | + | 0 | 0 | 0 | 0 |
| Narok | 4 | 3 | 1 | 72.0 | - | - | - | - |
| Nyamira | 0 | 0 | 0 | 0 | - | - | - | - |
| Taita Taveta | 0 | 0 | 0 | 0 | 0 | 0 | 0 | 0 |
| Vihiga | 0 | 0 | 1 | + | - | - | - | - |
| *indicates a statistically significant (p<0.05) relative reduction in mean intensity  +indicates an increase rather than relative reduction in mean intensity  -indicates areas where survey for *S. haematobium* was not undertaken | | | | | | | | |
